# Health and the House

**Published:** 1919-04-26

**Authors:** 


### HEALTH AND THE HOUSE.

### FROM A CORRESPONDENT.

Health is one of the greatest assets of a nation, and on a nation's houses its health largely depends. Insanitary houses, lacking in air and light, are the source of much of the disease that afflicts civilised peoples, and though a good deal of improvement has been effected in the last few decades, yet there are still streets in great cities on whose houses we might write "all health abandon ye who enter here"; there are still slums where the tubercle bacillus prospers, and where healthy minds and bodies cannot be bred. Even if we burned down all the bad irremediable houses, we should still have to plan to build better ones, and the disappointing thing is that even after centuries of building, and after scores of Housing Conferences and Housing Committees, we do not seem to have advanced very much in the science and art of building small houses, at once hygienic, convenient, and cheap.

Happily, however, there seems reason to believe that an architect who, for the last five years, has been working at the problem of building workmen's houses, has discovered a cheaper and more hygienic method of building and planning than has been hitherto devised. He finds that the bungalow house on one floor, and that the semi-detached house of two floors, are both wasteful of building material, and he comes to the conclusion that it is best to build in quadruple blocks, each block consisting of four flats, two on a ground floor and two on an upper floor, each flat being a complete, self-contained, five-roomed house.

Further economy he effects by a patent triangular wing which renders compactness possible without sacrifice of air and light, and still further economy by a new method of building. He makes four central chimney breasts the legs, so to speak, of the building, for he makes these breasts support the main weight of the roofing and flooring, after the fashion of a four-legged table with its legs moved near its centre. This method of building effects economy of material both in direct and indirect ways. It renders possible a patented method of central heating and of central ventilation, and permits an arrangement of the rooms which economises space. It also takes the weight of roofing and flooring off the walls, which are therefore exposed to little strain, and can be made of thin sheets of cement or other suitable material. In these simple, yet original, ways, so much structural material can be saved that a five-roomed house can be built with about 70 per cent. of the bricks required to build in the ordinary way a house of equal available cubic capacity. The patented system of central ventilation by air flues parallel with the chimney flues ensures a plentiful supply of pure air; but if any inmate of the house require still more fresh air, each house has an open-air balcony where a bed may be put. Since each house is a flat all its rooms are on one floor, and this naturally facilitates housekeeping. Both on the economic and on the hygienic side the architect seems to have made a valuable contribution to the science and art of house-building, and, if he can build a house with 70 per cent. of the bricks usually required, he will save the nation many millions of money.

There are still, however, a good many sanitary sides of building that require more study. It is not sufficient to have a house well lit, well ventilated, and clean, if the material of which it is built may itself harbour disease, and it is difficult to believe that the present plan of covering the floors of a house with boards in whose interstices dust and germs must accumulate can be hygienic. Equally difficult is it to give hygienic approval to the plan of covering lath-and-plaster walls with paper, which is liable to become a medium for the growth of moulds and microbes. In these days of substitutes and industrial inventions it should surely be possible to devise some preparation of waterproof cellulose—some dense, smooth, shining substance which would offer neither food nor foothold to germs, and which might be used both for walls and floors. Whatever big housing schemes are carried out in the future it is to be hoped that both the hygienic and economic sides of the building question will be carefully considered.

